# Comparing ^13^C methyl and deuterated methyl isotopic labeling for the quantification of methyl cellulose patterns using mass spectrometry

**DOI:** 10.1007/s00216-023-04622-w

**Published:** 2023-03-03

**Authors:** Sarah Schleicher, Gavin O’Connor, Petra Mischnick

**Affiliations:** 1 Institute of Food Chemistry, Technische Universität Braunschweig, Schleinitzstr. 20, 38106 Braunschweig, Germany; 2 Department of Biochemistry, Physikalisch-Technische-Bundesanstalt, Bundesallee 100, 38116 Braunschweig, Germany; 3 Department of Biochemistry and Bioinformatics, Technische Universität Braunschweig, Rebenring 56, 38106 Braunschweig, Germany

**Keywords:** ^13^CH_3_ versus CD_3_- isotope labeling, Liquid chromatography-electrospray ionization mass spectrometry, Quantitative mass spectrometry, Substituent distribution, Methyl cellulose, Oligosaccharide ethers

## Abstract

The methyl substitution along and among the polymer chains of methyl cellulose (MC) is commonly analyzed by ESI-MS after perdeuteromethylation of the free-OH groups and partial hydrolysis to cello-oligosaccharides (COS). This method requires a correct quantification of the molar ratios of the constituents belonging to a particular degree of polymerization (DP). However, isotopic effects are most pronounced for H/D since their mass difference is 100%. Therefore, we investigated whether more precise and accurate results could be obtained for the methyl distribution of MC by MS of ^13^CH_3_ instead of CD_3_-etherified *O*-Me-COS. Internal isotope labeling with ^13^CH_3_ makes the COS of each DP chemically and physically much more similar, reducing mass fractionation effects, but at the same time requires more complex isotopic correction for evaluation. Results from syringe pump infusion ESI-TOF-MS with ^13^CH_3_ and CD_3_ as isotope label were equal. However, in the case of LC-MS with a gradient system, ^13^CH_3_ was superior to CD_3_. In the case of CD_3_, the occurrence of a partial separation of the isotopologs of a particular DP resulted in slight distortion of the methyl distribution since the signal response is significantly dependent on the solvent composition. Isocratic LC levels this problem, but one particular eluent-composition is not sufficient for a series of oligosaccharides with increasing DP due to peak broadening. In summary, ^13^CH_3_ is more robust to determine the methyl distribution of MCs. Both syringe pump and gradient-LC-MS measurements are possible, and the more complex isotope correction is not a disadvantage.

**Graphical abstract:**

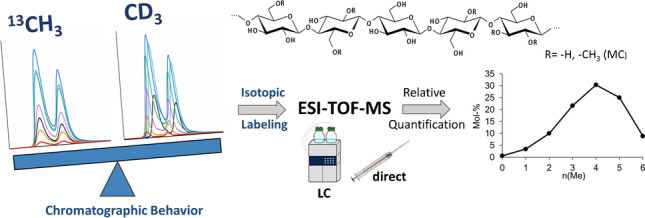

**Supplementary Information:**

The online version contains supplementary material available at 10.1007/s00216-023-04622-w.

## Introduction

On quantity basis alone, cellulose ethers are the most important cellulose derivatives. The annual production of carboxymethyl-, methyl-, and hydroxyalkylcellulose is about 420.000 t/a [1]. Due to their rheological, emulsifying, thickening, and film-forming properties, they have a wide range of applications in the food, cosmetic, and pharmaceutical as well as textile and building material industries. Their characteristics significantly depend on the molar mass, type of substituents, and the degree of substitution (DS). Beyond the average DS, the distribution of substituents on the positions 2, 3, and 6 in the glucosyl units and on higher hierarchical levels has an impact on their properties [2].

**Fig. 1:**
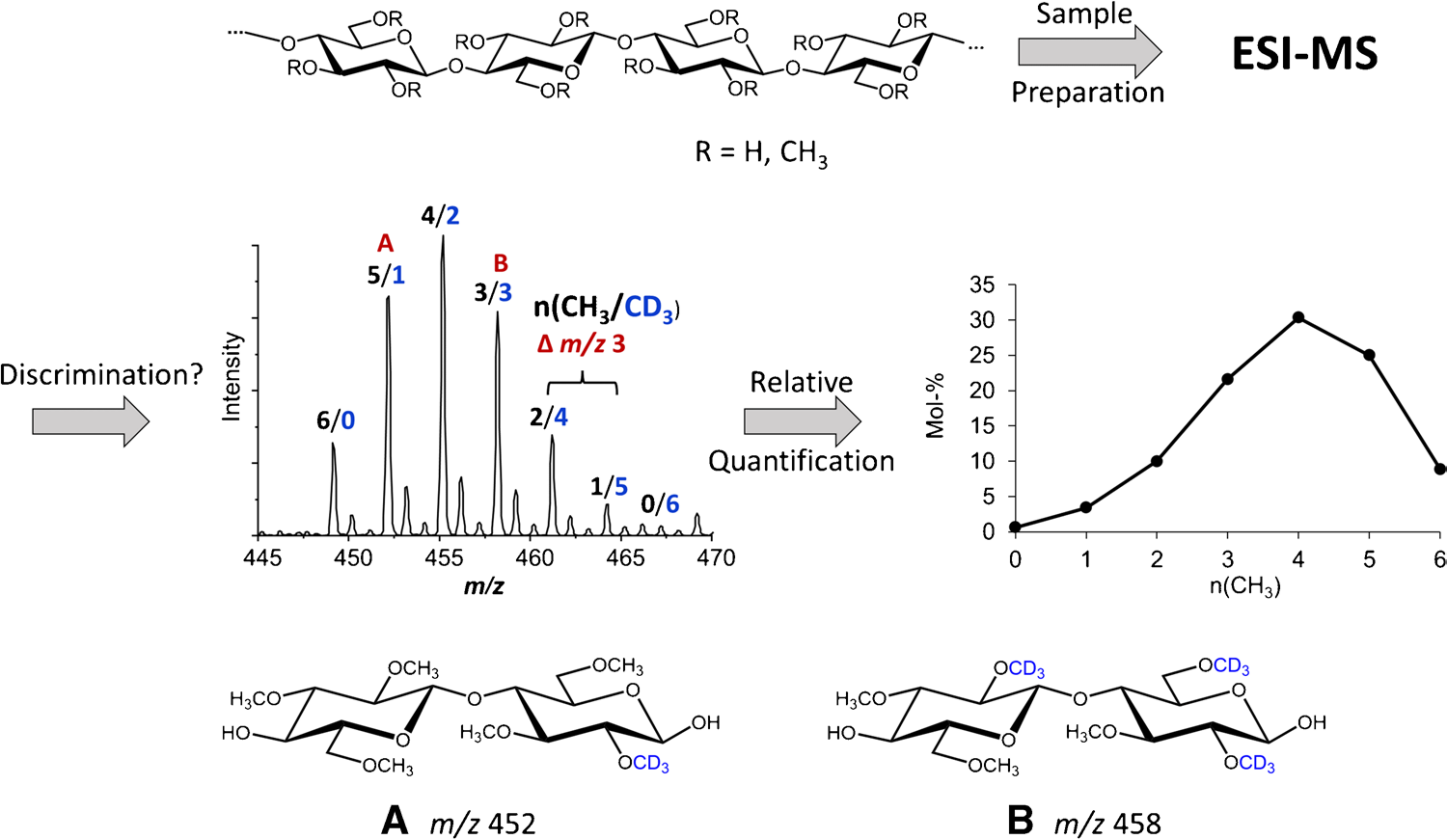
Scheme of determination of the substituent distribution of methylcellulose along and over the polymer chains by ESI-TOF-MS. Structures for A and B (Na^+^-adducts) are examples out of the possible regioisomers with the same total number of Me and Me-*d*_*3*_ and thus the same *m/z*

For determining the distribution pattern along and over the polymer chains, mass spectrometry (MS) is the method of choice. In the case of methylcellulose (MC), the free OH-groups are currently peralkylated with deuterated methyl iodide (CD_3_I), and the sample is randomly hydrolyzed to a complex mixture of cellooligosaccharides (COS). *O*-Me-*O*-Me-*d*_*3*_-COS are measured as sodium adducts [M+Na]^+^ in the positive mode [3–5] or after reductive amination with *m*-amino benzoic acid (*m*ABA) in the negative mode [4–7]. In order to evaluate the methyl substitution profile for each particular degree of polymerization (DP), the relative molar composition of all isotopologs of the formula (*O*-Me)_n_(*O*-Me-*d*_*3*_)_m_-COS (with n + m = 3⋅ DP), each of them comprising several constitutional isomers, is quantified (Fig. 1) [4, 5, 8]. So, it has to be ensured that all COS within a particular DP show the same efficiencies with respect to ionization in electrospray, transportation, mass analysis (for instance when using an ion trap (IT) or time of flight (TOF) mass analyzers), and detection. In short, no mass fractionation effects should occur if the correct mole ratios are to be determined. By using CD_3_ as internal isotope labeling, the chemical and physical differences of the COS with different numbers of methyl groups are reduced, and the ∆*m/z* of the consecutive COS of a particular DP is reduced from 14 to 3, i.e., from 42 to 9/glucosyl unit [9].

The extent and type of deviation of the so obtained substitution profiles of the individual DPs from the calculated statistical distribution indicate various types of heterogeneity of the cellulose ether, for instance, a DS gradient within the material and/or small amounts of poorly activated and thus scarcely derivatized cellulose, bimodality in blends, or even block-like structures [3, 5, 8, 10–12]. The method used is a type of isotope dilution mass spectrometry (IDMS). IDMS analysis can be used as a primary method of analysis, providing a direct unbroken chain of analysis anchored to the international system of units (SI). An isotopically labeled standard that should behave identically to the target analyte is added to the sample prior to preparation [13]. This means any source of bias during sample preparation (for instance, extraction yield, kinetics of derivatization) and MS analysis (matrix effects, ionization, etc.), can be prevented [13–15]. The most commonly used isotopes are non-exchangeable ^2^H, ^13^C and ^15^N, respectively. Widely practiced is the introduction of three to eight ^2^H or ^13^C atoms or a combination of both to separate the isotopologs of the isotopically enriched material from that of the unenriched material on a mass spectrometer [16]. While apparently simple, some requirements have to be considered regarding the implementation and the choice of the internal standard, as described in more detail in the literature [14, 15] and the guideline of IDMS [17].

In some incidences, isotope effects may be observed. Isotope effects describe a different chemical or physical behavior of isotopologs caused by their mass difference [18]. Li et al. showed that ^15^*N*-labeled melamine forms additional fragment ions compared to ^13^*C*-labeled melamine and natural melamine (kinetic isotope effect), resulting in an incorrect quantification [19]. Itoh et al. showed that the quantification of polycyclic aromatic hydrocarbons by IDMS leads to different results depending on whether a ^13^C- or ^2^H-labeled standard is used due to a different behavior during pressurized liquid extraction, but could also occur during SPE cleanup or selective sorptive losses [20].

Isotope effects are most pronounced for H/D, since their mass difference is 100%, and are largely caused by differences in vibrational frequencies. Due to the higher mass of D, the zero-point vibrational energy of a C-D linkage is lower and the amplitude of vibrations smaller, causing a reduction in bond length and consequently a smaller van der Waals radius and polarizability, compared to C-H [18]. As a result, the deuterated isomer is less retarded on a reversed phase column. The interaction of a molecule with the stationary phase depends on the binding energy. In the case of C-H, the frequency of the stretching vibration (wave number about 2900 cm^−1^) is higher than for C-D (about 2100 cm^−1^). This causes a stronger attraction between the C-H bond and the stationary phase. In general, the more H atoms are replaced by D atoms, the better is the separation between deuterated and non-deuterated isomers when using chromatographic separation [21]. Due to their relatively low cost and ease of preparation, deuterated internal standards are more commonly used. However, a different chromatographic behavior between the deuterated standard and the analyte negates the advantages of IDMS over other standards since matrix effects and ion suppression effects are no longer compensated as Wang et al. showed for the determination of carvedilol enantiomers in human plasma [22]. Consequently, deuterated standards are not always the best choice of internal standard. The relative mass difference between ^12^C and ^13^C or ^14^N and ^15^N, respectively, is significantly smaller; therefore, less isotope effects are expected, and these are preferable to labeling with deuterium [23].

Recently, we reported the comprehensive assessment of the accuracy of methyl pattern analysis by direct infusion ESI-IT-MS of deuteromethylated *O*-Me-COS. Measurements of model substances showed that mass fractionation effects occur within the DP, if the measurements are not performed under conditions optimized for each individual *m/z* range of interest [9].

When samples are separated and introduced to the mass spectrometer using liquid chromatography instead of syringe pump infusion, the chromatographic separation of the isotopologs resulted in a further potential source of bias. Therefore, the question arises whether more accurate results can be obtained with ^13^C methyl as internal label. On the one hand, the isotopologs and constitutional isomers of a particular DP would become chemically and physically more uniform. Furthermore, the peralkylation with^13^C methyl iodide (^13^CH_3_I) (∆*m/z* 1) instead of CD_3_I (∆*m/z* 3) would result in a narrower *m/z* range/DP and thus also less potential mass discrimination or fractionation effects during MS analysis. In the case of low resolution mass spectrometers, signal overlap caused by-products of the alkylation (under or overmethylation) or with doubly charged analytes of larger DPs with singly charged ones of half the DP (e.g., [DP6+2Na]^2+^ and [DP3+Na]^+^) can be avoided. Further advantages are a larger S/N ratio due to the distribution of the total intensity over a narrower *m/z* range. This could be particularly advantageous at the margins of the distribution profiles (see Fig. 1). However, at the same time, a more complex correction due to the natural ^13^C content of the analytes becomes necessary for ^13^C labeled analytes, which might negate these advantages.

Therefore, we investigated whether more precise and accurate results could be obtained for the methyl distribution of MC by MS of ^13^CH_3_ instead of CD_3_-etherified *O*-Me-COS. Two MCs, differing in DS, were investigated by ESI-TOF-MS. Both, syringe pump infusion and LC measurements were performed.

## Materials and methods

### Materials

With the exception of DMSO (≥99.5% for synthesis) and trifluoroacetic acid (TFA) (≥99.9%), purchased from Roth, as well as acetic acid (≥99.8%, LC-MS quality) (HOAc) from VWR Chemicals, all other chemicals were purchased from Sigma-Aldrich/Merck with the following purity: deuterated iodomethane (CD_3_I) (≥99.5% D), ^13^C-labeled iodomethane (^13^CH_3_I) (≥99.0% ^13^C), sodium hydroxide pellets, sodium borohydride granulate, and methyl lithium solution (1.6 M in diethyl ether).

The following solvents were used: toluene (Fisher Chemicals), acetone (Sigma-Aldrich/Merck), methanol (MeOH), and acetonitrile (ACN) (Riedel de Haen). For ESI-MS measurements, LC-MS grade was used; for all other applications, HPLC-grade was used.

Both MC samples, MC1 (DS_GC_ 1.29 - alditol acetate method [24]) and MC2 (DS_GC_ 1.96 - alditol acetate method), had been provided by former DOW Wolff Cellulosics GmbH, Bomlitz, Germany.

### Instrumentation

MS studies were performed on a timsTOF (Bruker Daltonics, Bremen, Germany) with the trapped ion mobility mode disabled. Ion source parameters for syringe pump infusion: nitrogen was used as dry gas (4 L min^−1^, 200 °C) and nebulizer gas (1 bar), capillary 2.7 kV, end plate offset 500 V. Tune parameters: deflection 1 delta 60 V, funnel 1 RF 300 Vpp, funnel 2 RF 200 Vpp, multipole RF 200 Vpp, ion energy 5 eV. All other parameters were adjusted depending on the measured DP/ mass range of the analyzed isotopologs and are shown in Table 1. Mass spectra were recorded with a scan range from *m/z* 200 to 1300 and a spectra rate of 5 Hz. The standard deviation of mass calibration was usually  ≤0.14 ppm. Resolution  ≥30,000 FWHM.

**Table 1:** Tune measurement parameters for the individual DPs of *O*-Me/*O*-Me-*d*_*3*_-COS (also used for *O*-Me/*O*-^13^C-Me-COS)

DP	*m/z *[M+Na]^+^	IsCID energy (eV)	Low mass (*m/z*)	Collision energy (eV)	Collision RF (Vpp)	Transfer time (µs)	Pre pulse storage (µs)
2	449–467	0	300	7	700	60	14
3	653–680	0	200	7	1000	70	17
4	857–893	50	200	7	1200	100	18
5	1061–1106	80	200	10	1500	100	22

For LC, the ESI-TOF-MS was coupled to an Agilent LC system equipped with a binary pump (1290 series) and autosampler (1290 series). The following parameters were changed compared to syringe infusion: dry gas (8 L min^−1^, 220 °C), nebulizer gas (2.5 bar), capillary voltage (4.5 kV). Mass Spectra were recorded with a scan range from *m/z* 100 to 1400 and a spectra rate of 7 Hz.

### Synthesis of isotopic labeled cellulose ethers

MC1 and MC2 were ^13^C-methylated according to a modified Hakomori method using Li-dimsyl in DMSO as base, which was prepared by addition of the required volume of methyllithium solution to the same volume of dry DMSO under nitrogen [12, 25]. Afterwards, the samples were dialyzed against water and freeze-dried. Completeness of alkylation was checked by the absence of OH absorption  >3000 cm^−1^ by ATR-IR spectroscopy. If complete alkylation had not been achieved, the alkylation was repeated with less equivalents of the reagents.

The deuteromethylation of MC1 and MC2 was initially performed according to the method of Ciucanu and Kerek [26]. In case of incomplete alkylation, the reaction was carried out again according to the modified Hakomori method referred above.

### Partial hydrolysis

Hydrolysis was performed for CD_3_-labeled MC with 2 M TFA, for ^13^CH_3_ cellulose ethers with 1 M TFA (final concentrations). Therefore, the approach for ^13^CH_3_ is given in parentheses in the following:

The permethylated MC (4 mg) was weighted in a 2 mL V-vial and swollen in 1.7 mL (1.85 mL) of a mixture of water and acetone (50/50, v/v) overnight. At the next day, 0.3 mL (0.15 mL) TFA (conc.) was added, and the sample was heated at 120 °C for 22 min (43 min). Afterwards, the samples were cooled to room temperature, and the aqueous acid was removed in a stream of nitrogen at ambient temperature by co-destillation with toluene and finally evaporated to dryness. The residue was dissolved in 2 mL 90% MeOH (c = 2 mg mL^−1^).

For syringe pump infusion experiments, the partial hydrolysates were diluted to 0.01 mg mL^−1^ (c ~ 1–2·10^−5^ M) in 90% MeOH and for LC experiments to 0.05 mg mL^−1^ (c ~ 0.5–1·10^−4^ M) in 80/20 H_2_O/ ACN (v/v).

### Determination of the substituent distribution in COS by ESI-MS

For the determination of the substituent distribution, the *O*-Me/*O*-Me-*d*_*3*_-COS and *O*-Me/*O*-^13^CH_3_-COS were infused continuously by a syringe pump at a flow rate of 200 µL h^−1^ into the ESI source. The samples were measured several times, adjusting the optimal instrumental parameters for each particular DP, and the TIC was recorded for 5 min. (For details, see the “Instrumentation” section.) The measurements were repeated on two additional days.

In addition, LC measurements were performed on a RP-C_18_-column (Phenomenex Kinetex RP18; 100 × 2.1 mm, 2.6 µm) at 40 °C and a flow rate of 0.2 mL min^−1^. A linear gradient system was used, consisting of water (A) and ACN (B) with 1% HOAc as eluent, starting at 90% A to 40% A within 10 min, and injection volume was 4–6 µL. As for syringe infusion experiments, for each particular DP, the COS were measured at the respective optimal measurement parameters three times (see the “Instrumentation” section).

The evaluation of all methyl distribution profiles was performed by Bruker Daltonics Data Analysis software. Only sodium adducts were considered, beside these potassium and ammonium adducts were also observed. This was possible, since the isotopologs showed the same complexation behavior. For syringe pump measurements, an average mass spectrum of the TIC was generated for each DP, and the mass intensities were used for evaluation, whereas for LC measurements, the area of the EIC ([M+Na]^+^ ±7 ppm) of the isotopologs was used for evaluation. For the relative molar quantification of all constituents belonging to one DP, the mass intensities (syringe pump) or the areas of the EICs (LC-MS) were corrected for noise and their isotopic compositions (for more details, see the “Results and discussion: Syringe pump infusion” section). Finally, the intensities/ areas were summed up and normalized to 100%. Normally, each sample was measured three times on different days, and the normalized methyl distributions of these days were averaged and re-normalized.

In order to consider all signals contributing to a particular (*O*-CH_3_)_m_-(*O*-R)n-COS in the quantitative evaluation, the isotopolog distribution of each constituent was calculated with the program *Isotope Distribution Calculator* (IDCalc, by Michael J. MacCoss, Department of Genome Sciences, University of Washington), which also allows consideration of the isotopic purity of the isotopic-labeled alkylating reagents in its calculation.

For the subsequent comparison of the two sets of isotopic-labeled COS (syringe pump) or any comparison of two distributions (e.g., from LC-gradient MS and syringe pump infusion or from within multiple/repeated measurements of one sample to express its uncertainty), the DS of the respective normalized methyl distribution was determined as well as the root mean square (RMS) value to express the overall deviation between the distributions obtained by labeling with ^13^CH_3_ and CD_3_, respectively, to reference data, which are specified in the text:

1 $$DS=\frac{\sum n\left(substitued\ OH\ groups\right)\cdot {c}_{i}}{100 \cdot DP}$$

*n(substituted OH groups)*: number of substituted OH groups
*c*_*i*_: molar amount in mol%
*DP*: degree of polymerization

2 $$RMS=\sqrt{\frac{\sum_{i=1}^n\left(\;x_i(CD_3)-x_i\left({}^{13}{CH_3}\right)\right)^2\;\;\;\;\;\;}{n-1}}$$

*x*_*i*_* (CD*_*3*_*)*: experimental methyl distribution for CD_3_ labeling
*x*_*i*_* (*^*13*^*CH*_*3*_*)*: experimental methyl distribution for ^13^CH_3_ labeling
*n*: number of data points

### Reduction of COS

In order to obtain glucitol-terminated COS, no longer forming α-and β-anomers, 1 mg in 500 µL of the partially hydrolyzed MC was transferred into a 500 µL V-vial, and the solvent was removed under a stream of nitrogen. Afterwards, the sample was re-dissolved in 500 µL of a NaBH_4_ solution (c = 6 µmol mL^−1^ in H_2_O) and heated at 60 °C for 90 min. After cooling to room temperature, the solvent was removed in a stream of nitrogen. The residue was dissolved in 500 µL ACN (c = 2 mg partial hydrolysate mL^−1^), and completeness was checked by ESI-IT-MS with syringe infusion (Bruker HCT Ultra ETD II) [9]. For LC experiments, the samples were dissolved to 0.05 mg mL ^−1^ (c ~ 0.5–1·10^−4^ M) in 80/20 H_2_O/ ACN (v/v).

## Results and discussion

### Syringe pump infusion

To assess the accuracy of the measurements of differently isotope-labeled COS, proven reference data are required. We recently generated such reference data for the deuterated MCs used in this study by using uniform COS standard solutions of defined concentration and optimizing the instrumental settings of an ESI-IT-MS [9]. Thus, in the first step, we verified that these reference data can be reproduced by ESI-TOF-MS after optimization of the measurements conditions. As expected, this was the case (ESM Fig. S1). The relative root mean square (RMS) for MC2 was between 0.31 and 0.52, on average 0.41 ± 0.10, without any DP-related trend. The RMS for the repeated measurements of the sample ranged from 0.03 ± 0.01 to 0.08 ± 0.02. In the second step, we investigated if the more complex isotope correction algorithms, required when using ^13^CH_3_ labeling for the determination of substituent distributions of MCs, leads to an observable and significant bias which would therefore negate any advantages over CD3 labeling.

Before presenting the results, the more complex isotope correction for ^13^CH_3_ compared to CD_3_ as label should be explained in more detail: for the evaluation, an average mass spectrum is generated, and the relative intensities of all constituents/isotopologs belonging to a particular DP are corrected for their isotopic compositions, i.e., the total intensity of an isotopolog is calculated by adding the theoretical contribution of the higher natural isotopologs to the main signal. In the case of CD_3_ with ∆*m/z* 3, overlap with the next main signal only starts at [M+3]. This contribution is, for instance, 4.7% when compared with the main peak of the largest evaluated COS, DP5. However, on the decreasing intensity side of the MS profile (corresponding to decreasing DS), the relative contribution of these natural isotopologs of the preceding signal to the next isotopolog signal increases and should not be neglected.

**Fig. 2:**
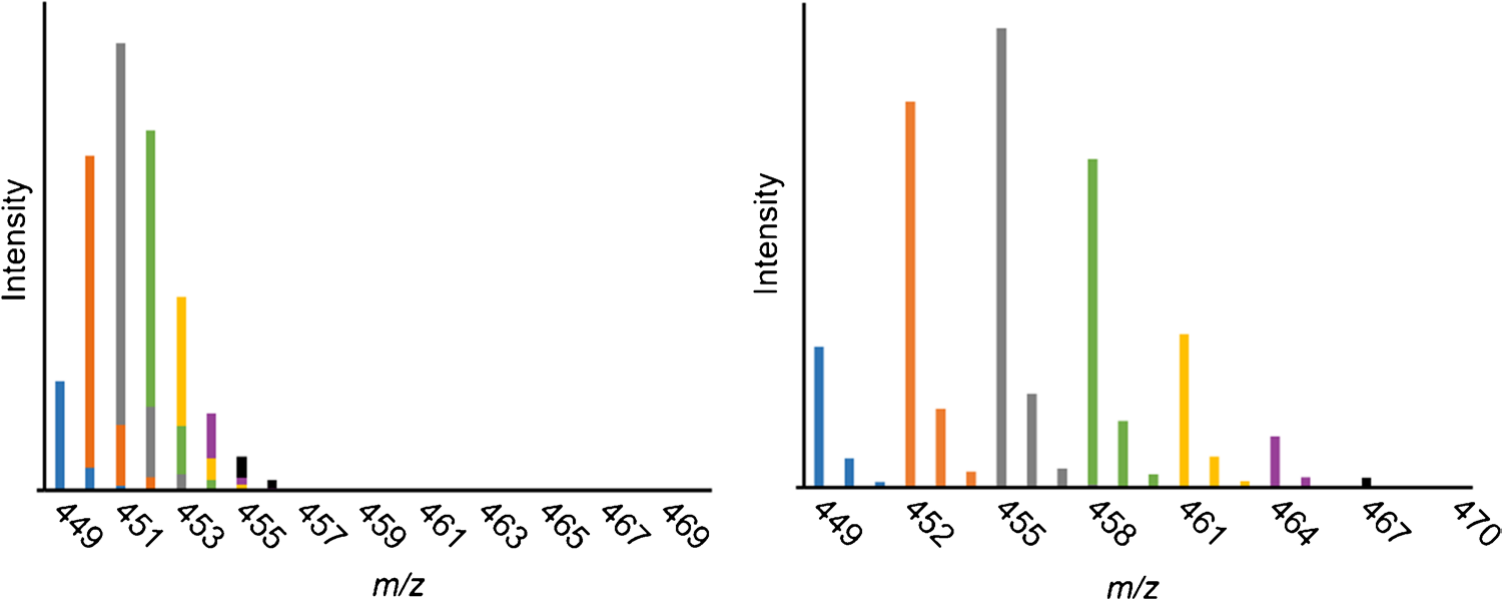
Schematic representation of the mass spectra obtained for [M+Na]^+^ of DP2 of MC2 in case of internal ^13^CH_3_ (left) and CD_3_ labeling (right). The color code demonstrates the intensity distribution and overlay for each *O*-(^13^CH_3_)_n_ and *O*-(CD_3_)_m_-cellobiose, respectively, of interest

In the case of ^13^CH_3_ labeling with ∆*m/z* 1, the overlap of the natural isotopologs with the signal of the next labeled isotopolog is much more remarkable as shown in Fig. 2 for DP2 as an example. Each natural isotopolog of a certain (*O*-CH_3_)_n_(*O*-^13^CH_3_)_m_-COS overlaps with the main signal of (*O*-CH_3_)_n-1_(*O*-^13^CH_3_)_m+1_-COS and subsequent signals, respectively. Since the theoretical natural isotopolog contribution caused by the preceding signal is subtracted from the subsequent main peak, the *S/N* of the measurement is of great importance for the quality of the corrections. Furthermore, depending on the isotope content (purity) of the ^13^CH_3_I applied, superposition of [M-1] signals with the previous isotopolog signal might be relevant. However, in our case, purity was 99.0%, and thus this correction was not taken into account. Finally, the intensities of each DP are normalized to 100%.

Beside the reagent, the natural content of ^13^C in cellulose as well as from the methyl chloride used for the synthesis of MC is also of interest. The natural ^13^C content varies with the carbon source but was found to be negligible. MCs are usually produced from cellulose of cotton plants (C3 plant). During the biosynthesis, there is a depletion of ^13^C due to kinetic isotope effects; as a result, the ^13^C isotope abundance is 1.08% instead of the natural abundance of 1.11% atom. Overall, the isotopic correction for ^13^CH_3_ in plants is more complex and error-prone than for CD_3_ [27].

**Fig. 3:**
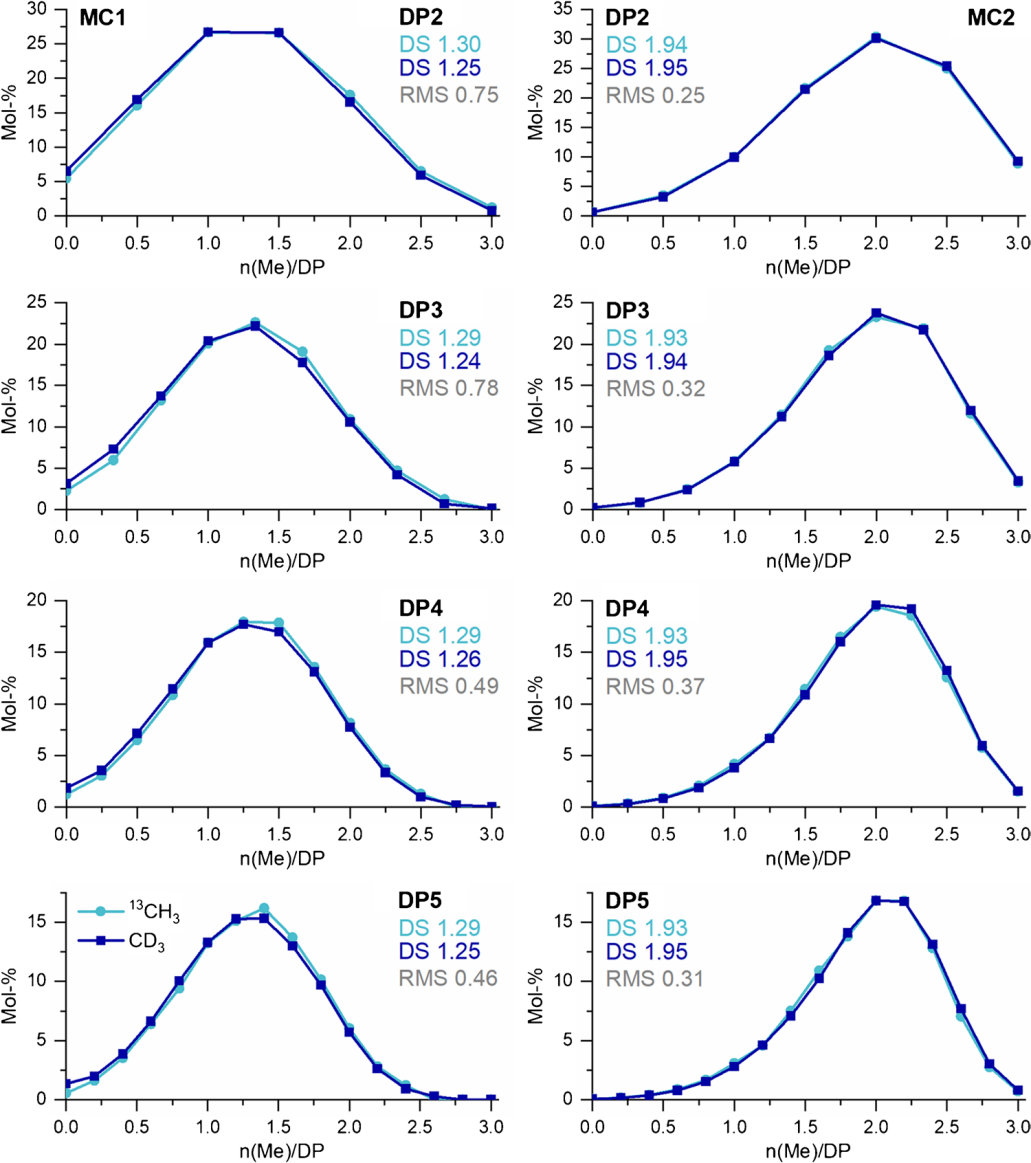
Comparison of the methyl distribution obtained by ESI-TOF-MS for MC-derived COS labeled with ^13^CH_3_ or CD_3_, respectively. Left: MC1(DS_GC_ 1.29). Right: MC2 (DS 1.96). Samples were applied by syringe pump infusion. Instrumental settings were adjusted according to the DP/ *m/z* range of the analyzed isotopologs (see “Materials and methods: Instrumentation” section). The deviation of the methyl distribution of the two labeling methods is given as root mean square (RMS). *n* = 3

In order to check, whether the more complex isotope correction counteracts the advantages of ^13^CH_3_ over CD_3_ when used as the label, the COS derived from MC1 (DS_GC_ 1.29) and MC2 (DS_GC_ 1.96), each isotopically labeled either with CD_3_I or with ^13^CH_3_I, respectively, were introduced by syringe pump infusion to the ESI-TOF-MS. As already mentioned above, it is important for the relative quantification that no discrimination effects occur during the analysis. Therefore, for each particular DP, the COS were measured under optimized conditions by recording the TIC, mainly the pulse times were adjusted according to the increase in the mass of the isotopologs (see Table 1). Figure 3 shows the results. In the left column, the methyl distributions obtained for MC1; in the right column, the corresponding results for MC2 are presented for both types of isotopologs, respectively. To express the overall deviation of the substituent distribution obtained from the ^13^C-labeled COS from the CD_3_-reference, the RMS was calculated. Syringe pump infusion eliminates chromatographic mass fractionation effects. Thus, only the impact of the more complex isotope correction is reflected, as outlined above.

For MC2, the profiles are in very good agreement for both types of isotopologs. The RMS for ^13^CH_3_ referred to the reference data (CD_3_) is between 0.25 and 0.37. Accordingly, the DS/DP, which should be in agreement with the average DS of the MC, differs only slightly between the two samples of 1.93–1.94 (^13^CH_3_) and 1.94–1.95 (CD_3_), respectively. For MC1, the deviation of the methyl distribution calculated for the two types of isotopologs is more pronounced, already visible in deviations of the DS/DP. For ^13^CH_3_, it ranges from 1.29 to 1.30, for CD_3_ from 1.24 to 1.26, but without any trend. The RMS observed for this was between 0.46 and 0.78. Since the average DS_GC_ of MC1 is 1.29, the results are even in better accordance with the ^13^CH_3_-labeled COS. However, it should be emphasized at this point that the alkylation of the low DS MC1 is less reproducible than for MC2; therefore, higher RMS are obtained.

In summary, both labeling methods are suitable for determining the substituent distribution of MCs. The more complex isotope correction for ^13^CH_3_ did not turn out to be a significant source of error. Therefore, in the following the data from the syringe infusion, ESI-TOF-MS of the respective isotopologs will be used as reference data for all further measurements.

### Application by LC-ESI

#### LC gradient measurement

**Fig. 4:**
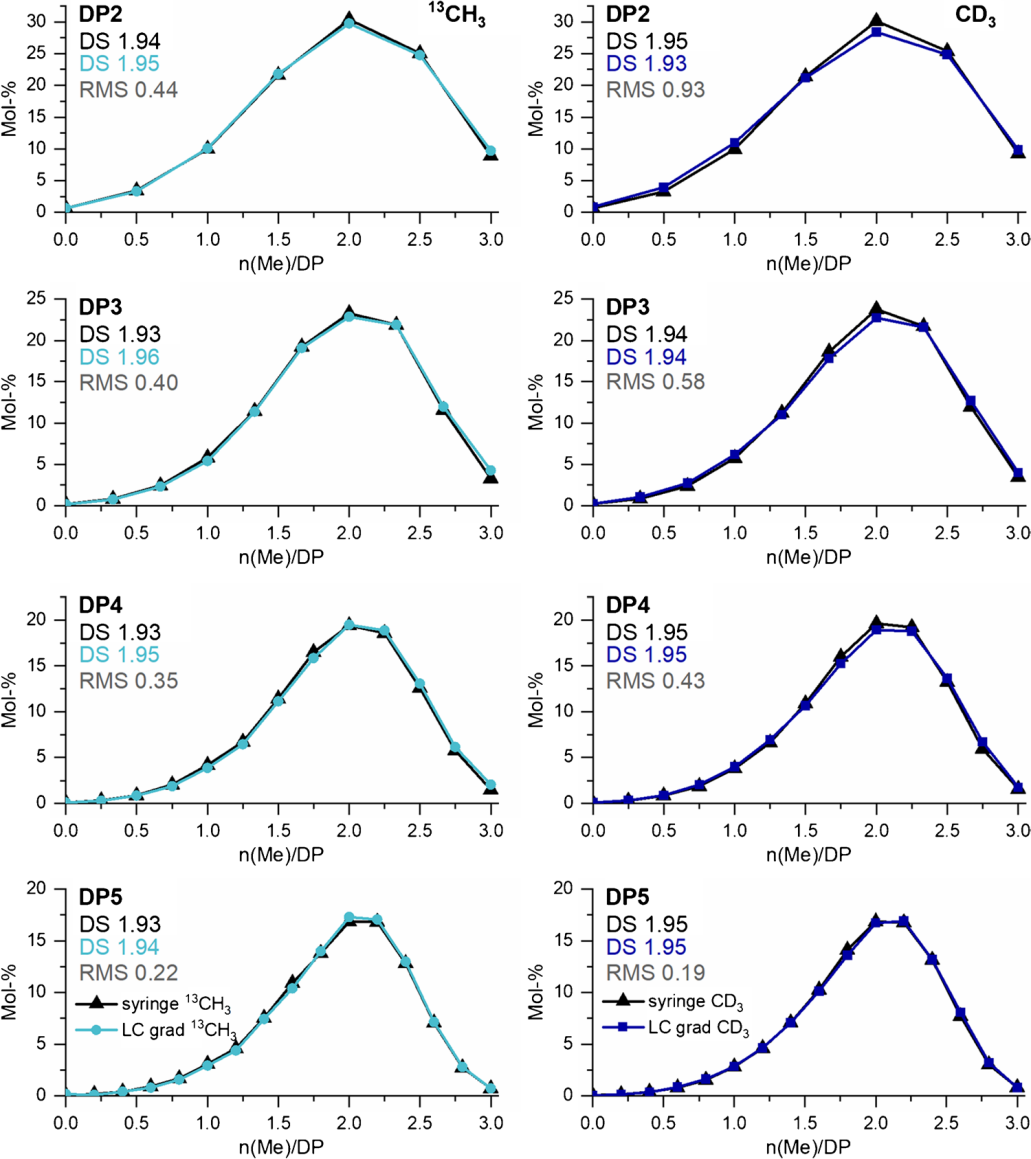
Methyl distribution in COS derived from MC2 (DS_GC_ 1.96) obtained by LC-ESI-TOF-MS on a RP18 column with gradient elution, compared to those obtained by syringe pump infusion of the same sample. Left: ^13^CH_3_-, right: CD_3_-labeled COS. Measurement parameters were adjusted depending on the measured DP/ *m/z* range of the analyzed isotopologs (see “Materials and Methods: Instrumentation” section). The deviation of the LC-gradient-results from the syringe infusion reference data is given as (RMS). *n* = 3

In contrast to syringe pump infusion, LC-MS measurements can be more easily automated which is advantageous for larger number of samples. Therefore, we also investigated LC-MS as a method of analysis. With the ESI-IT-MS used previously [9], the low resolution did not enable the isobaric signals of the target analytes [M+Na]^+^ and the commonly accompanying [M+NH_4_]^+^ ions to be resolved from each other using LC-MS. With the high resolution ESI-TOF-MS, this is no longer a problem. Figure 4 compares the methyl distributions obtained by LC-ESI-TOF-MS for the two types of isotopologs, ^13^CH_3_ (left) and CD_3_ (right) of MC2. The corresponding distributions from the syringe pump measurements are added as reference data. To express the overall deviation of the methyl distributions obtained by LC-MS from the reference data, again the RMS was calculated. For the results of MC1, see ESM Fig. S2.

The DS value for the individual samples is almost constant over the DP. However, it is obvious that the methyl distribution profiles derived from the gradient-LC-MS data of the *O*-CD_3_-labeled COS clearly deviate from the reference data (MC2 RMS: 0.19–0.93, MC1 RMS: 0.19–0.76, see ESM Fig. S2). For both deuteromethylated MCs, the deviation is most pronounced for DP2 and was reproducible (averaged RMS within DP2: 0.18 ± 0.04 (MC2) and 0.13 ± 0.05 (MC1)). It should be emphasized that for the *O*-CD_3_-COS, there is a clear decreasing trend of the RMS for the LC-MS-results compared to the reference data with the DP. Such DP-related trends are an additional indicator of measurement bias. Since the COS of one sample should all bear the same information, the results should be independent of the DP. In contrast, the distributions obtained for the ^13^C-isotopologs again very well agree with the reference profile without any RMS trend. The RMS was always  ≤0.44 for the individual DPs for both MCs. In summary, when applied by gradient-LC, ^13^CH_3_ is superior to CD_3_ in terms of accuracy.

**Fig. 5:**
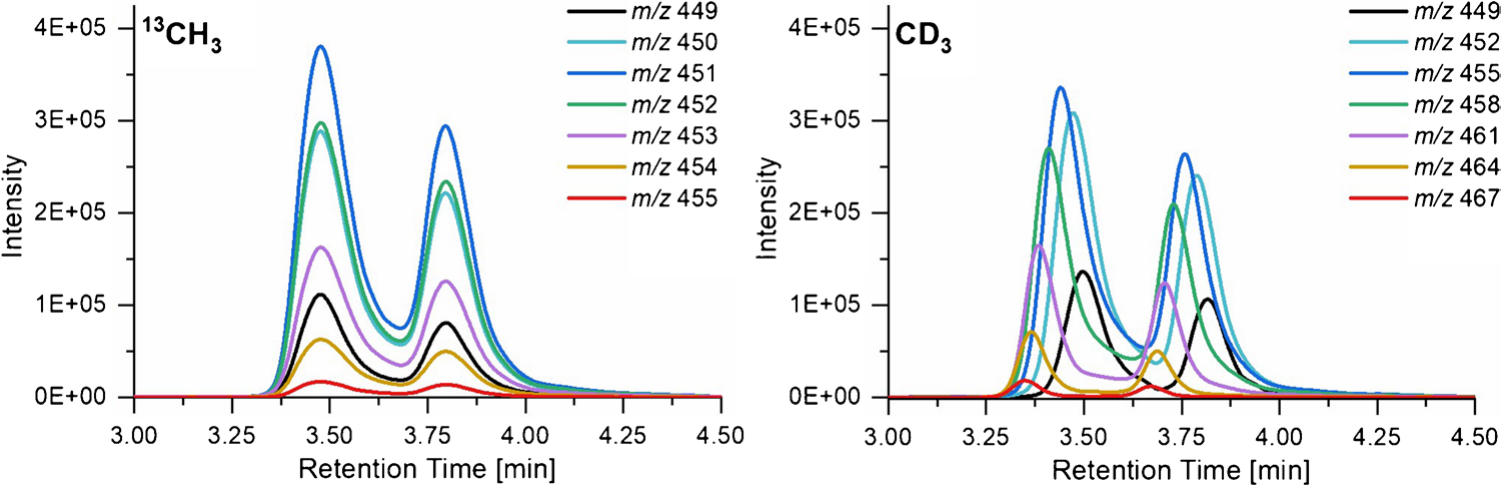
EIC of the *m/z* of the isotopologs belonging to DP2 of (left) ^13^CH_3_ - and (right) CD_3_-labeled COS derived from MC2. Separation was performed on a RP18 column at 40 °C with H_2_O (A) and ACN (B) as eluents, each with 1%HOAc. Starting with 90% A, decreasing to 40% A within 10 min. The first peak is assigned to the α-anomer, the second to the β-anomer

So, why do the results differ for LC-ESI-MS, while they are in agreement for the same sample solutions when applied by syringe pump? The only difference between the two types of isotopologs is their chromatographic behavior, which is not existing in the syringe pump infusion measurements. As can be seen in Fig. 5 exemplified for DP2, in the case of ^13^CH_3_ labeling (left), all isotopologs elute simultaneously from the column as is also described in the literature for other ^13^C-labeled standards [23, 28]. The two peaks/DP correspond to the α- and the β-anomers due to mutarotation at the reducing end of the COS. At each time, the molar ratio of the isotopologs is the same. However, in the case of CD_3_ labeling (right), partial separation with respect to the number of CD_3_ groups occurs for each anomer. As has been outlined in the introduction, the more CD_3_ (or more generally, the more D), and consequently the less CH_3_, the less retarded is the isotopolog on the RP phase [21]. Consequently, the molar ratio of the isotopologs changes with the elution time within each anomer of a particular DP. It is known from the literature that different chromatographic behavior in IDMS can lead to erroneous results, e.g., due to matrix suppression effects [22, 23]. In our case, we are dealing with “pure substances,” so that matrix effects can probably be neglected.

However, COS ethers for the determination of substitution patterns are far more complex than samples of simple metabolites quantified via IDMS. Within a particular DP, we observe isotopologs with different numbers of CH_3_ (m) and isotopic methyl groups (n). And each of these isotopologs comprises a huge number of regioisomers increasing with DP and with a maximum of possibilities for n = m. This constitutional isomerism causes additional peak broadening. Furthermore, all these constituents exist in two diastereomeric forms, the α- and the β-anomers which are clearly separated (Fig. 5). Finally, the situation is even more dynamic since these stereoisomers mutate during the separation. Rate of mutarotation of a particular carbohydrate depends on the column temperature which was set at 40 °C in our experiment to achieve an appropriate peak form. Since at this temperature, mutarotation kinetics is in the order of magnitude of chromatographic processes, a plateau between anomers can sometimes be observed. This plateau is caused by those molecules which have occasionally been in the α- and in the β-form, respectively [29]. Beside the temperature, the equilibrium is also affected by the solvent composition and its pH [30]. Consequently, the equilibrium of the anomers is continuously adjusted during the chromatographic separation, because of the gradient system.

All these aspects make quantification more difficult. Ideally, for LC measurements, the sample should not change during the analysis but show a minimum of complexity.

To reduce the complexity of the sample, the CD_3_-labeled samples were reduced with NaBH_4_, to the corresponding glucitol-terminated COS (COS-ol), so that mutarotation no longer takes place, and measured with the gradient system as well as an isocratic system (70/30, A/B) at 40 °C. The results are shown in Fig. S3. The distributions measured with gradient-LC still significantly deviate from distributions for DP2 and DP3 of the non-reduced samples, received by syringe pump infusion, whereas the results fit very well, if the samples were measured in an isocratic system. The RMS for the latter was lower for each individual DP. Hence, most probably the change in solvent composition during elution in the gradient system causes different ionization efficiencies for the isotopologs and thus the observed bias in LC gradient and measurements.

### LC-ESI-MS under isocratic conditions and influence of the solvent composition

The experiments with the reduced sample had pointed out that the occurrence of two stereoisomers for each isotopolog is not responsible for the deviation of LC results from the syringe infusion results. To confirm that the change of measurement conditions (e.g., solvent composition during elution and thus the ESI-process) within each group of isotopologs is a more critical factor, the non-reduced COS were also measured with the isocratic system. Furthermore, the samples were equilibrated in the isocratic solvent mixture (without the addition of HOAc) at 40 °C.

**Fig. 6:**
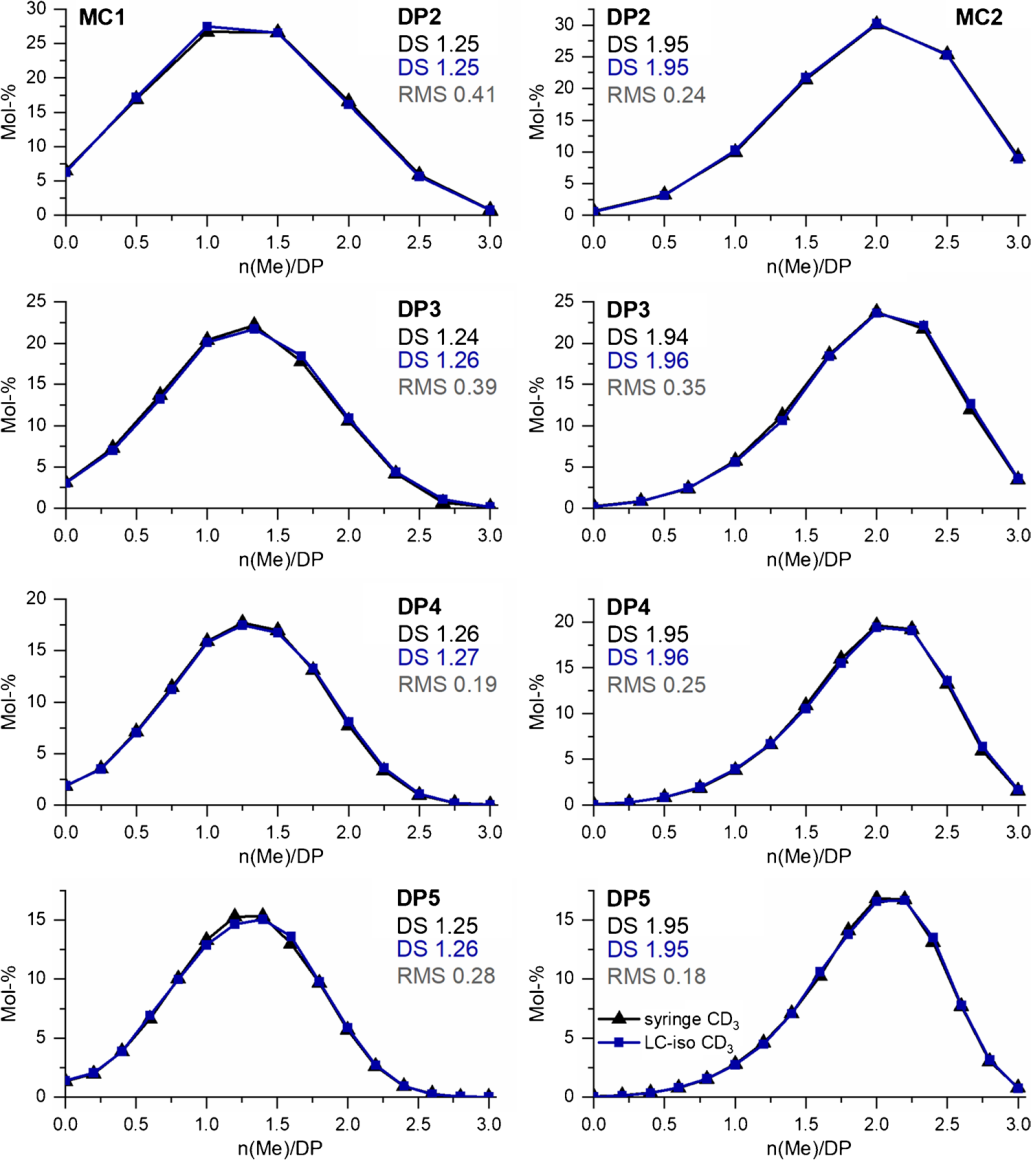
Methyl distribution in COS after labeling with ^13^CH_3_ and CD_3_ of (left) MC1 (DS_GC_ 1.29) and (right) MC2 (DS_GC_ 1.96), respectively, measured by LC-ESI-TOF-MS on a RP18 column with an isocratic system (70/30 H_2_O+1% HOAc/ ACN+1%HOAc, v/v) compared to syringe pump infusion of the same sample (reference data). Instrumental setting was adjusted according to the DP/ *m/z* range of the analyzed isotopologs (see “Materials and method: Instrumentation” section). RMS is referred to reference data, *n* = 3

The results are shown in Fig. 6. and compared to the reference data. The distributions are now in perfect agreement with the reference data for both MCs. For MC1, the RMS was always  ≤0.41 and for MC2  ≤0.35 without any RMS trend and thus very close to those observed for the LC measurement for ^13^CH_3_ labeling (Fig. S2 and Fig. 4, left).

The results confirm that the critical reason for the slight distortion of the methyl distribution profile derived from the gradient-LC-MS analysis is the elution order of the isotopologs and consequently their ionization at different solvent compositions, although the retention time differences between the fully methylated and deuteromethylated isotopologs are just 5–17 s, depending on the DP. Separation in α- and β-anomers expands the problem since each group of these stereoisomers elutes in another solvent composition range. However, as seen from the reduction experiments, this effect is obviously marginal related to the basic phenomenon. In the gradient system, the ACN portion (B) increases by 1.8–4.2% in absolute terms during elution of the individual isotopologs belonging to one DP. The eluent composition changes most for DP2 (for individual isotopologs from 3.0 to 4.2%), for which the deviation of the methyl distribution from the syringe pump results was the largest. For comparison, for the components belonging to DP5, the change is only between 1.8 and 2.9%. But maybe more important, the relative change and thus the impact on the ESI process is much in the beginning, when the water content is still high, than for the later eluting COS, where the ACN content approximates 50%. As is known from the literature, the ionization efficiency depends on the properties of the analyte, for instance, its surface activity and complexing properties, e.g., for sodium, furthermore the composition of the solvent including concentration of electrolytes, its surface tension and evaporation energy, and finally the instrumental settings of the MS [31–33]. In our previous study, we demonstrated by syringe pump infusion that the isotopologs belonging to a particular DP are ionized with the same efficiency at constant solvent composition [9]. However, we also observed that the extent of formation of sodium, potassium, and ammonium adducts depends on the solvent composition and can consequently change during a gradient separation. As mentioned above, in the syringe infusion measurements, it is sufficient to consider only the sodium adducts.

Furthermore, the composition of the solvent in relation to the chosen ESI capillary voltage has a decisive influence on the signal intensity. Solvents with a low surface tension and high volatility, such as methanol or acetonitrile, and their mixtures with water are particularly suitable. Rapid evaporation leads to increased fission rates of the charged droplets (Coulomb explosion) and more efficient release of analyte ions. Pure water, on the other hand, is less suitable due to its high surface activity, low volatility, and thus poor spray stability, resulting in low intensities [31, 32]. Therefore, for LC-MS, the intensities of the compounds eluting at a higher ratio of the organic solvent usually increase [31]. A high amount of water requires a larger capillary voltage/electric field. For a stable spray, the applied voltage, the flow rate, and the conductivity and surface tension of the solvent must be balanced. If the voltage is set too high, electric discharge may occur, which leads to unstable ion currents and decreased response [31, 32]. In our case, the capillary voltage of 4.5 kV was set to a relatively high value due to the high water content at the beginning of the chromatographic separation. In summary, the solvent composition has a decisive influence on the signal intensity due to the points mentioned above; this can lead to bias for LC-MS analysis if chromatographic separation occurs. Isocratic measurement can overcome this effect; however, due to peak broadening, gradient-LC-MS of ^13^CH_3_ labeled COS is recommended as the more robust method.

## Conclusion

We could show that ^13^CH_3_ and CD_3_ are equivalent as isotopic labeling when the samples are applied by syringe pump infusion and thus measured at identical and constant conditions for all analytes. The more complex isotopic correction for ^13^C did not cause any distortion of the profiles. However, when samples were introduced to the MS using a gradient-LC- separation method, results for the ^13^CH_3_-labeled COS were superior to CD_3_-etherified ones for which an, albeit small, but significant deviation from the reference data was observed. This deviation was largest for DP2 and decreased with DP. By further experiments, i.e., reduction of COS to COS-ol to focus the α-and β-anomers in one peak, and by isocratic LC-MS, it was proved that the chromatographic behavior of the CD_3_-labeled COS is responsible for the observed bias. In case of CD_3_-labeled-COS, the isotopologs are partially separated when applied by LC, with the higher-deuterated ones of each anomer being less retained on the RP-column. In contrast, in the case of ^13^CH_3_ labeling, all isotopologs belonging to one anomer elute simultaneously. Consequently, the molar ratio of the isotopologs changes during the elution time of each anomer of a particular DP as well as the solvent composition during elution and ESI.

In conclusion, we could show that ^13^CH_3_-labeling is much more robust with respect to sample application and instrumental settings to determine the methyl distribution of MCs since all isotopologs and the isotopomers of each isotopolog behave equally. The more complex isotope correction does not cause any disadvantage.

## Supplementary Information

Below is the link to the electronic supplementary material.

Supplementary file1 (DOCX 395 KB)
