# Supplementary material for: Comparing 13C methyl and deuterated methyl isotopic labeling for the quantification of methyl cellulose patterns using mass spectrometry
Source: Anal Bioanal Chem. 2023 Mar 3;415(10):1817–28. doi: 10.1007/s00216-023-04622-w (PMC10050035; doi:10.1007/s00216-023-04622-w)
Supplement: Supplementary file 1 — Supplementary file1 (DOCX 395 KB) [file 216_2023_4622_MOESM1_ESM.docx]

*Analytical and Bioanalytical Chemistry*

## Electronic Supplementary Material

**Comparing ^13^C methyl and deuterated methyl isotopic labeling for the quantification of methyl cellulose patterns using mass spectrometry**

Sarah Schleicher^a^*, Gavin O’Connor^b,c^, Petra Mischnick^a^*

^a^ Institute of Food Chemistry, Technische Universität Braunschweig, Schleinitzstr. 20,

38106 Braunschweig, Germany

^b^ Department of Biochemistry, Physikalisch-Technische-Bundesanstalt, Bundesallee 100, Braunschweig 38116, Germany

^c^ Department of Biochemistry and Bioinformatics, Technische Universität Braunschweig, Rebenring 56, 38106 Braunschweig, Germany

*Corresponding author: Petra Mischnick, p.mischnick@tu-braunschweig.de

Sarah Schleicher, s.schleicher@tu-braunschweig.de

ORCID, Sarah Schleicher: 0000-0003-0159-5141

ORCID, Gavin O’Connor: 0000-0002-7812-6717

ORCID, Petra Mischnick: 0000-0002-8313-3313

**A) Comparison of the results obtained for MC2 by ESI-TOF-MS and to ESI-Ion Trap-MS by syringe pump infusion**

For a relative quantification of COS, derived from isotopically labeled methyl cellulose (MC), by MS, it is important that no mass fractionation effects occur. Therefore, for the ESI-TOF-MS measurements, the instrumental settings were optimized for each DP, primarily by adjusting the pulse time to the *m/z* range of the isotopologs of the respective DP. The comparison of the results obtained by syringe infusions to an ESI-TOF-MS with proved reference data obtained by ESI-Ion Trap (IT)-MS [1] showed that the selected measurement parameters were appropriate. Fig. S1 compares the methyl distribution of CD_3_-labeled COS from MC2 (DS_GC_ 1.96), measured by ESI-IT-MS and ESI-TOF-MS. To express the overall deviation of the data derived by ESI-TOF-MS from the ESI-IT-MS-data, the RMS (root mean square) value was calculated (see body text, equation 2).

The obtained methyl distributions are in very good agreement, the RMS ranged from 0.31 to 0.52. The optimized ESI-TOF-MS settings are consequently suitable for a discrimination-free determination of the methyl distribution.


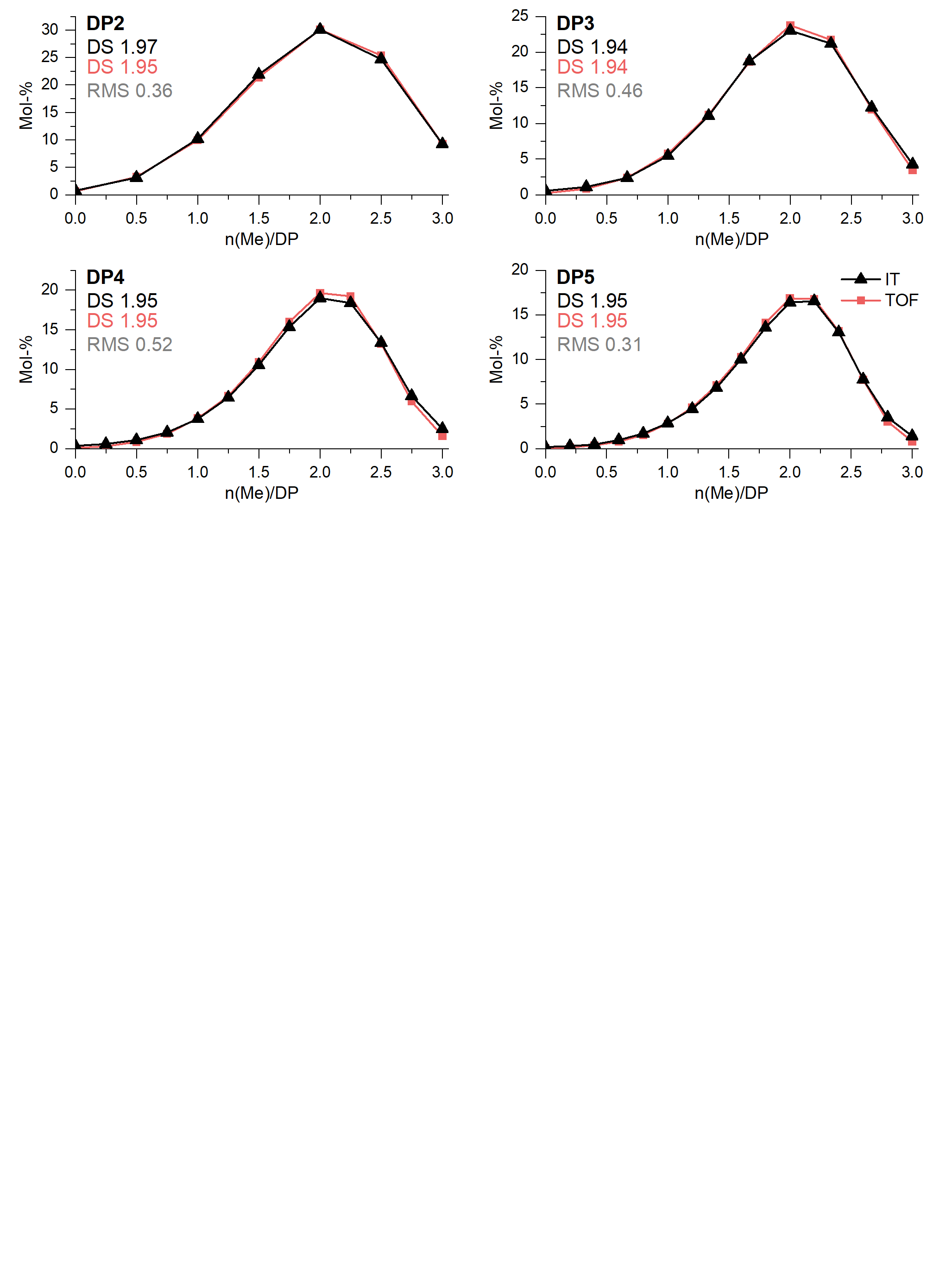


**Fig. S1** Comparison of the obtained methyl distributions in MC-derived COS after labeling with CD_3_. MC2 (DS 1.96) measured by ESI-Ion Trap-MS (black) and ESI-TOF-MS (red) by syringe pump infusion. The overall deviation of the methyl distribution between ion trap (IT) and TOF measurement is given as root mean square (RMS). n=3

[1] Schleicher S, Lottje IR, Mischnick P. Impact of instrumental settings in electrospray ionization ion trap mass spectrometry on the analysis of multi-CH_3_-/CD_3_-isotopologs in cellulose ether analysis: a quantitative evaluation*.* Anal Bioanal Chem. 2022;414:1279–1296*.*

10.1007/s00216-021-03767-w

**B) Gradient-LC-ESI-TOF-MS of COS derived from isotopically labeled MC1**

The LC-separation was performed on a RP18 column at 40 °C with H_2_O and ACN as eluents, each with 1 % HOAc, starting with 90 % A to 40 % A within 10 min. The results for ^13^CH_3_ (left) and CD_3_-labeled COS (right) from MC1 (DS_GC_ 1.29) are shown in Fig. S2. The confirmed data obtained by ESI-TOF-MS with syringe pump infusion of the respective samples are included as reference data. To express the overall deviation between the gradient-LC MS result and the reference data, the RMS is calculated. For the discussion of the results see body text.

**

**

**Fig. S2** Methyl distributions in COS, derived from MC1 (DS_GC_ 1.29) after labeling with ^13^CH_3_ (left) and CD_3_ (right), respectively. Results from gradient-LC and syringe pump infusion ESI-TOF-MS are compared. LC: RP18, gradient system of A: H_2_O and B: ACN, both with 1% HOAc. Solvent for syringe pump infusion: 90 % MeOH. Measurement parameters were adjusted depending on the *m/z* range of the DP of interest (see body text, *Instrumentation*). The deviation of the methyl distribution for both types of isotopologs by gradient LC compared to syringe pump infusion is given as root mean square (RMS). n= 3

**C) LC-ESI-TOF-MS measurement of glucitol-terminated CD_3_-labeled COS (COS‑ol)**

In order to minimize the complexity of the CD_3_-labeled COS, the samples were reduced with NaBH_4_ to the corresponding glucitol-terminated COS (COS-ol). Thus, α- and β-anomers and their mutarotation do no longer exist. COS-ol samples were measured in the LC gradient system as well as in an isocratic system of 70/30 A/B (A: H_2_O B: ACN, each 1% HOAc). In Fig. S3, the corresponding methyl distributions are compared with those of the syringe infusion-MS measurements of the non-reduced sample (reference data). To express the overall difference between the methyl distributions obtained by LC-MS and the reference data, the root mean square value (RMS) was calculated.





**Fig. S3** Methyl distribution profiles obtained for *O*-Me-*O*-Me-*d_3_*-COS-ol by ESI-TOF-MS. Left: MC1 (DS_GC_ 1.29); Right: MC2 (DS_GC_ 1.96). Samples were applied by gradient-LC (start: 90% A and 40% A within 10 min), and an isocratic system (70% A), respectively. The separation was performed on a RP18 column at 40 °C and H_2_O (A) and ACN (B), each with 1% HOAc, as eluents. Results obtained for the corresponding α,β-*O*-Me-*O*-Me-*d_3_*-COS, applied by syringe infusion are included in the graphics as reference data. The overall deviation of the methyl distributions obtained for each type of LC application from the syringe pump measurement are given as root mean square (RMS). n= 3

For both MC1 (DS_GC_ 1.29) and MC2 (DS_GC_ 1.96), the distribution of the reduced samples measured under isocratic LC-conditions (70/30 A/B) was in good agreement with the distribution obtained from the syringe infusion-MS of the non-reduced sample. The root mean value was between 0.23 and 0.39 for MC1 and between 0.28 – 0.41 for MC2. On the other hand, if the reduced samples are measured under gradient conditions, the deviation is greater (RMS(MC1) 0.44 – 0.93; RMS(MC2): 0.38 – 0.92). Again, the difference was largest for MC1 for DP2 and DP3 and for MC2 for DP2.
